# Supplementary material for: Outcomes of Kidney Transplant Patients with Atypical Hemolytic Uremic Syndrome Treated with Eculizumab: A Systematic Review and Meta-Analysis
Source: J Clin Med. 2019 Jun 27;8(7):919. doi: 10.3390/jcm8070919 (PMC6679118; doi:10.3390/jcm8070919)

Online supplementary data 1

Search terms for systematic review.

Databases: Ovid MEDLINE

1. Kidney.mp
2. Renal.mp
3. exp kidney/
4. 1 OR 2 OR 3
5. Transplant.mp
6. Transplantation.mp
7. exp transplant/
8. exp transplantation/
9. 5 OR 6 OR 7 OR 8
10. 4 AND 9
11. Eculizumab.mp
12. 10 AND 11

Databases: EMBASE:

('kidney transplantation' OR 'kidney graft' OR 'kidney graft rejection') AND eculizumab

Database: Cochrane Databases

'eculizumab and kidney transplant in Title Abstract Keyword'

Supplement Table S1: Newcastle-Ottawa quality assessment scale of included studies in meta-analysis assessing use of eculizumab for treatment of aHUS on patients after kidney transplantation

Study	Selection				Comparability	Outcome			Total score
	Represent-ativeness	Selection of the non-exposed cohort	Ascertainment	Endpoint not present at start	Comparability (Confounding)	Assessment of outcome	Follow-up duration	Adequacy follow-up	
Legendre et al., 2017	*	*	*	*		*	*	*	7
Levi et al., 2017	*	*	*	*		*	*	*	7
Manani et al., 2017	*	*	*	*		*	*		6
Sheerin et al., 2016	*	*	*	*		*			5
Mallett et al., 2015	*	*	*	*		*	*	*	7
Matar et al., 2014	*	*	*	*		*	*	*	7
LeQuintrec et al., 2013	*	*	*	*		*	*	*	7
Zuber et al, 2012	*	*	*	*		*	*	*	7
Kocak, et al., 2015	*	*	*	*		*			5
Modelli de Andrade et al., 2017	*	*	*	*		*	*	*	7
Cavero et al., 2017	*	*	*	*		*	*	*	7
Zuber et al., 2017	*	*	*	*		*	*	*	7
Siedlecki et al., 2019	*	*	*	*		*	*	*	7

Notes: The Newcastle-Ottawa scale uses a star system (0 to 9) to evaluate included studies on 3 domains: selection, comparability, and outcomes. Star (*)= item presents. Maximum 1 star (*) for selection and outcome components and 2 stars (**) for comparability components. Higher scores represent higher study quality.

Supplement Table S2: Newcastle-Ottawa quality assessment scale of included studies in meta-analysis assessing use of eculizumab for prevention of aHUS on patients after kidney transplantation

Study	Selection				Comparability	Outcome			Total score
	Represent-ativeness	Selection of the non-exposed cohort	Ascertainment	Endpoint not present at start	Comparability (Confounding)	Assessment of outcome	Follow-up duration	Adequacy follow-up	
Levi et al., 2017	*	*	*	*		*	*	*	7
Sheerin et al., 2015	*	*	*	*		*			5
Matar et al. 2014	*	*	*	*		*	*	*	7
Zuber et al., 2012	*	*	*	*			*	*	7
Modelli de Andrade et al., 2017	*	*	*	*		*	*		6
Zuber et al., 2017	*	*	*	*		*	*	*	7
Bresin et al., 2013	*	*	*	*		*			5
Kumar et al., 2016	*	*	*	*		*	*	*	7
Favi et al., 2017	*	*	*	*	*	*	*	*	8
Yelken et al., 2017	*	*	*	*		*	*	*	7
Siedlecki et al., 2019	*	*	*	*		*	*	*	7

Notes: The Newcastle-Ottawa scale uses a star system (0 to 9) to evaluate included studies on 3 domains: selection, comparability, and outcomes. Star (*)= item presents. Maximum 1 star (*) for selection and outcome components and 2 stars (**) for comparability components. Higher scores represent higher study quality.

Supplementary Figure S1: Forrest plot (excluding studies with potentially included duplicate patients) evaluating for the incidence of aHUS recurrence among patients who received prophylactic eculizumab (The pooled estimated incidence 7.0% (95%CI: 2.5%-18.1%, I^2 =0%)).

Group by Type	Study name	Event rate	Lower limit	Upper limit	Z-Value	p-Value
Case series	Kumar et al	0.050	0.003	0.475	-2.029	0.042
Case series		0.050	0.003	0.475	-2.029	0.042
Cohort	Matar et al	0.100	0.006	0.674	-1.474	0.140
Cohort	de Andrade et al	0.167	0.010	0.806	-1.039	0.299
Cohort	Zuber et al (2)	0.029	0.004	0.177	-3.476	0.001
Cohort	Yelken et al	0.063	0.004	0.539	-1.854	0.064
Cohort	Favi et al	0.038	0.002	0.403	-2.232	0.026
Cohort		0.055	0.018	0.160	-4.733	0.000
Overall		0.055	0.019	0.146	-5.149	0.000

Statistics for each study

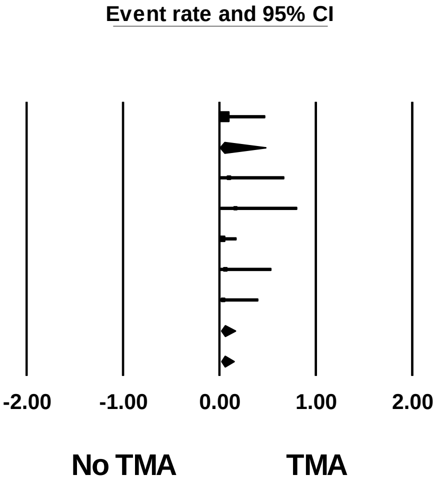

Supplementary Figure S2: Forrest plot (excluding studies with potentially included duplicate patients) evaluating for the incidence of allograft loss due to TMA among patients who received prophylactic eculizumab (The pooled estimated incidence 5.3% (95%CI: 2.4%-11.3%, I^2 =0%).

Study name	Event rate	Lower limit	Upper limit	Z-Value	p-Value
Matar et al	0.100	0.006	0.674	-1.474	0.140
de Andrade et al	0.167	0.010	0.806	-1.039	0.299
Kumar et al	0.050	0.003	0.475	-2.029	0.042
Yelken et al	0.063	0.004	0.539	-1.854	0.064
Favi et al	0.083	0.012	0.413	-2.296	0.022
Siedlecki et al	0.034	0.011	0.100	-5.692	0.000
	0.053	0.024	0.113	-6.837	0.000

Statistics for each study

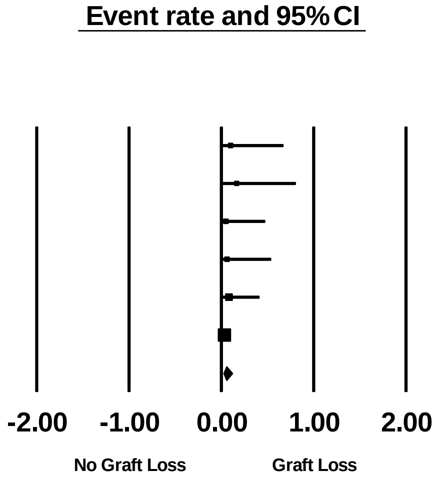

Supplementary Figure S3: Forrest plot (excluding studies with potentially included duplicate patients) evaluating for the incidence of allograft loss due to all causes among patients who received eculizumab treatment (The pooled estimated incidence 26.7% (95%CI: 13.0%-46.9%, I^2 =33%)).

Group by Type	Study name	Event rate	Lower limit	Upper limit	Z-Value	p-Value
Case series	Manani et al	0.833	0.194	0.990	1.039	0.299
Case series	Aigner et al	0.100	0.006	0.674	-1.474	0.140
Case series		0.421	0.017	0.968	-0.167	0.868
Cohort	Matar et al	0.667	0.154	0.957	0.566	0.571
Cohort	de Andrade et al	0.083	0.005	0.622	-1.623	0.105
Cohort	Mallett et al	0.667	0.154	0.957	0.566	0.571
Cohort	Kocak et al	0.100	0.006	0.674	-1.474	0.140
Cohort	Cavero et al	0.063	0.004	0.539	-1.854	0.064
Cohort	Siedlecki et al	0.240	0.166	0.333	-4.923	0.000
Cohort		0.259	0.123	0.466	-2.255	0.024
Overall		0.267	0.130	0.469	-2.230	0.026

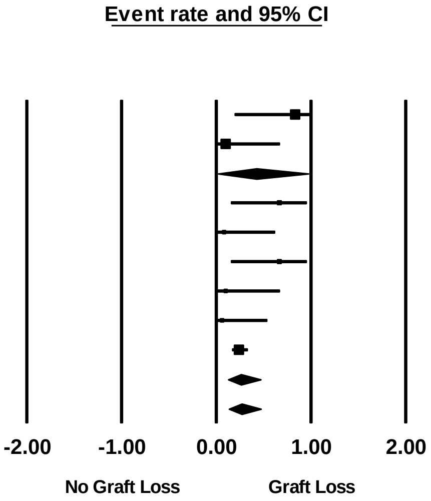

Supplementary Figure S4: Forrest plot (excluding studies with potentially included duplicate patients) evaluating for the incidence of allograft loss due to all causes among patients who received eculizumab treatment (The pooled estimated incidence 20.0% (95%CI: 9.1%-38.4%, I^2= 29%)).

Group by Type	Study name	Event rate	Lower limit	Upper limit	Z-Value	p-Value
Case series	Aigner et al	0.100	0.006	0.674	-1.474	0.140
Case series	Manani et al	0.833	0.194	0.990	1.039	0.299
Case series		0.421	0.017	0.968	-0.167	0.868
Cohort	Cavero et al	0.063	0.004	0.539	-1.854	0.064
Cohort	de Andrade et al	0.083	0.005	0.622	-1.623	0.105
Cohort	Kocak et al	0.100	0.006	0.674	-1.474	0.140
Cohort	Legendre et al	0.019	0.001	0.236	-2.781	0.005
Cohort	Mallett et al	0.333	0.043	0.846	-0.566	0.571
Cohort	Matar et al	0.333	0.043	0.846	-0.566	0.571
Cohort	Zuber et al (2)	0.353	0.168	0.596	-1.194	0.232
Cohort		0.189	0.083	0.375	-3.027	0.002
Overall		0.200	0.091	0.384	-2.976	0.003

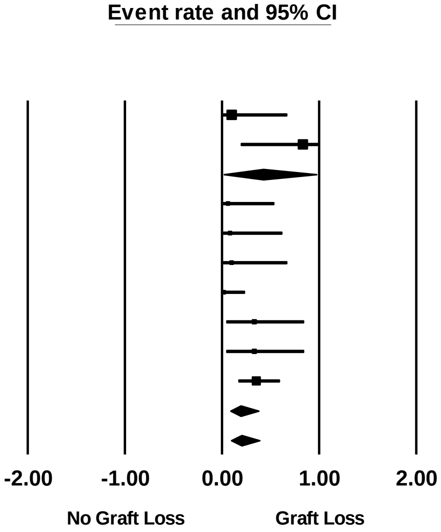

Supplement: Supplementary file 1 [file jcm-08-00919-s001.pdf]
